# Pneumonia or Kaposi Sarcoma: Beneath the Dyspnea With Non-compliance of HIV

**DOI:** 10.7759/cureus.30152

**Published:** 2022-10-10

**Authors:** Pooja Roy, Anusha Parisapogu, Harshita Agrawal

**Affiliations:** 1 Internal Medicine, Harlem Hospital Center, New York, USA; 2 Department of Laboratory Medicine and Pathology, Mayo Clinic, Rochester, USA; 3 Department of Infectious Diseases, Mayo Clinic, Rochester, USA

**Keywords:** dyspnea, pneumonia, hiv, kaposi sarcoma, pulmonary nodule

## Abstract

Human herpesvirus 8 (HHV-8) or Kaposi sarcoma herpesvirus is the cause of Kaposi sarcoma (KS), the most prevalent cancer related to acquired immune deficiency syndrome. About 90% of the time, KS is accompanied by cutaneous lesions; however, systemic illness can develop without cutaneous involvement. Today’s highly active antiretroviral therapy (HAART) era has seen a decrease in the prevalence of KS. In immunocompromised individuals, it may be challenging to differentiate between pneumonia and the clinical characteristics of pulmonary KS, which might make diagnosis more challenging. HAART is the first-line therapy for KS, and its usage has reduced the incidence of KS. Depending on how severe the illness is, systemic chemotherapy could be helpful. We report the case of a young man who presented with pulmonary symptoms in the presence of a pharyngeal mass and was later found to have bilateral pulmonary metastasis. Interestingly, this diagnosis was made in the absence of classic cutaneous lesions. The patient was counseled for quality of life with medication and intervention compliance, and a consultation with an oncologist was set up.

## Introduction

Moritz Kaposi, a Hungarian physician, originally characterized Kaposi sarcoma (KS) in its typical form in 1872 as an angio-proliferative disease [1-4]. A vascular lesion with low-grade malignant potential known as KS is associated with human herpesvirus 8 (HHV-8) infection. KS is a multifocal tumor that most often appears in mucocutaneous locations, such as the oropharyngeal mucosa, face, trunk, and lower limbs. Lymph nodes and visceral organs, particularly the respiratory and digestive systems, are also frequently affected by KS [1]. The World Health Organization (WHO) and the Centers for Disease Control and Prevention (CDC) both claim that KS is an acquired immune deficiency syndrome (AIDS)-defining illness [2,3].

According to the WHO GLOBOCAN data for 2018, there were 19,902 KS fatalities (13,117 men and 6,785 females) and 41,799 new KS diagnoses (28,248 males and 13,551 females) [5]. The CD4 count and the risk of AIDS-related KS are inversely correlated [3]. Based on the many populations it affects, there are four major epidemiologic subtypes of KS, namely, classic, which affects older males from the Mediterranean region more frequently; African endemic, which mostly affects young black men; immunosuppression-related; and AIDS-related. Most frequently, AIDS-related KS affects males who have intercourse with other men and are HIV positive (MSM). Hodgkin lymphoma, acute lymphocytic lymphoma, and solid organ cancers are also more common in KS patients. Additionally, plasmablastic lymphoma, primary effusion lymphoma, and multicentric Castleman disease linked to KS-associated herpesvirus (KSHV) are more common in KS patients [3]. Fever, joint discomfort, splenomegaly, lymphadenopathy, diarrhea, and exhaustion are some of the traditional signs and symptoms [5,6]. Patients with significant cutaneous disease, which is expected to advance to visceral involvement, are more likely to develop pulmonary KS [2]. A chest computed tomography (CT) scan was used in this case to detect pharyngeal KS that had spread to both lungs after pharyngeal lesions were noted. Even in the era of highly active antiretroviral therapy (HAART), this example demonstrates the significance of taking metastatic pulmonary KS into account as a differential diagnosis when patients with AIDS present with lung symptoms, especially if there is a history of non-compliance to HAART.

## Case presentation

A 31-year-old African American male with a history of HIV diagnosed one month prior with a current CD4 count 28 and viral load 44 came to our medical institution complaining of escalating exhaustion, lethargy, diarrhea, nausea, vomiting, and dyspnea on exertion for four days. He also had a moderate cough and yellowish sputum, but he denied experiencing any orthopnea, paroxysmal nocturnal dyspnea, chest discomfort, fever, shortness of breath, dizziness, syncope, recent travel, or any ill contacts.

He was hospitalized six months earlier due to fever, an ineffective cough, increasing dyspnea with exertion, and excessive perspiration but no concomitant hemoptysis or pleuritic discomfort. He had chronic complaints of throat irritation, a scratchy sensation on his tongue, and slight difficulty while swallowing food. However, he denied associated dysphagia or odynophagia. He was treated for *Pneumocystis jirovecii* pneumonia, oropharyngeal and esophageal *Candida*, and was advised to get tested for HIV and start definitive management. However, the patient refused despite counseling stating he had tested negative in the past and declined the consent for HIV testing; hence, he was discharged undiagnosed.

He smoked 10 cigarettes per day but quit one year ago. He denied the use of alcohol or other illicit substances. He endorsed being heterosexual and engaging in regular unprotected intercourse, including anal intercourse.

During this admission, he was noted to be emaciated and chronically ill-appearing with temporal wasting. He was lethargic, febrile, and tachycardic, with a heart rate of 124 beats/minute. He also tested positive for coronavirus disease 2019 (COVID-19) and was hypoxic with an oxygen saturation of 77% on room air. He had bilateral coarse crackles with bilateral pedal edema. His oral examination revealed white plaques in the throat and buccal cavity, indicative of a *Candida *infection. A mass projecting from the posterior pharynx was also visualized, associated with difficulty in swallowing and regurgitation along with submandibular adenopathy. A review of other systems revealed no abnormality.

**Figure 1:**
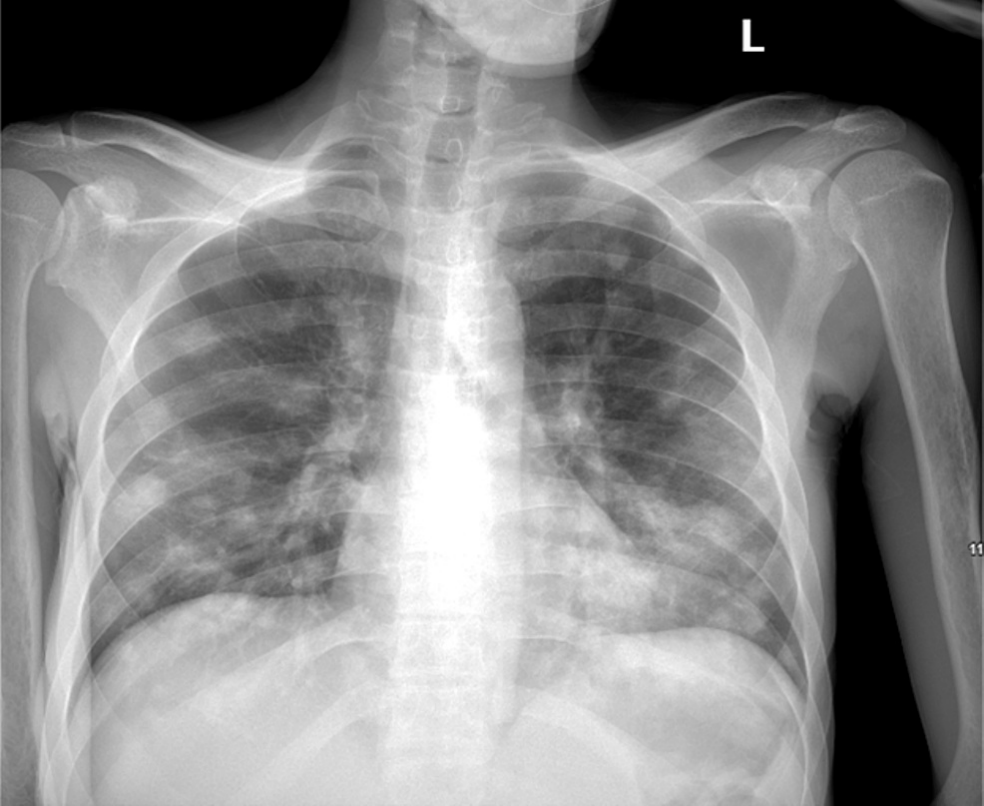
Chest X-ray showing bilateral patchy opacities.

His labs were significant for hemoglobin of 8.3 g/dl and hematocrit of 25.2%, with features of malnutrition (total protein 6, albumin 1.3). The chest X-ray revealed bilateral extensive patchy airspace opacities involving all lung fields, primarily involving bilateral lung bases, and interval increase compared to a prior study that was suggestive of an atypical infectious process and further need for malignancy evaluation (Figure 1).

**Figure 2:**
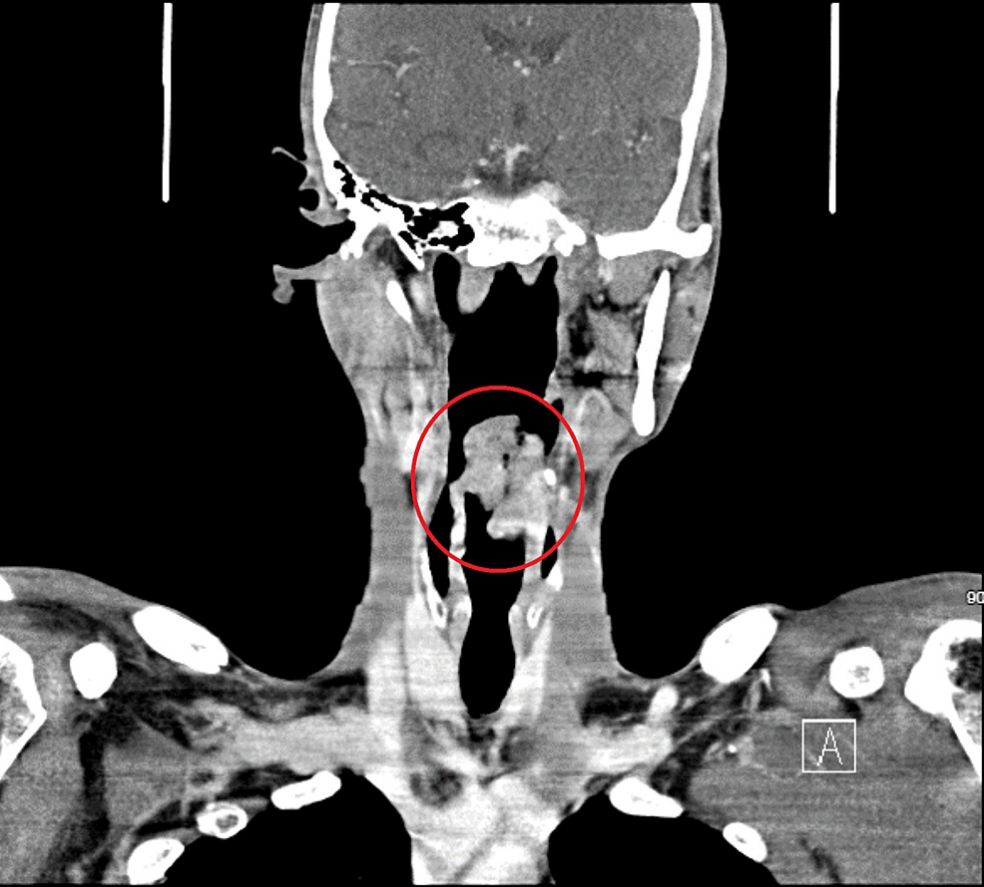
Computed tomography of the soft-tissue neck with contrast coronal section (lesion measuring 4.2 × 3.7 × 6.7 cm (AP, TR, SI) in the circle). AP: anteroposterior; TR: transverse; SI: superior to inferior

**Figure 3:**
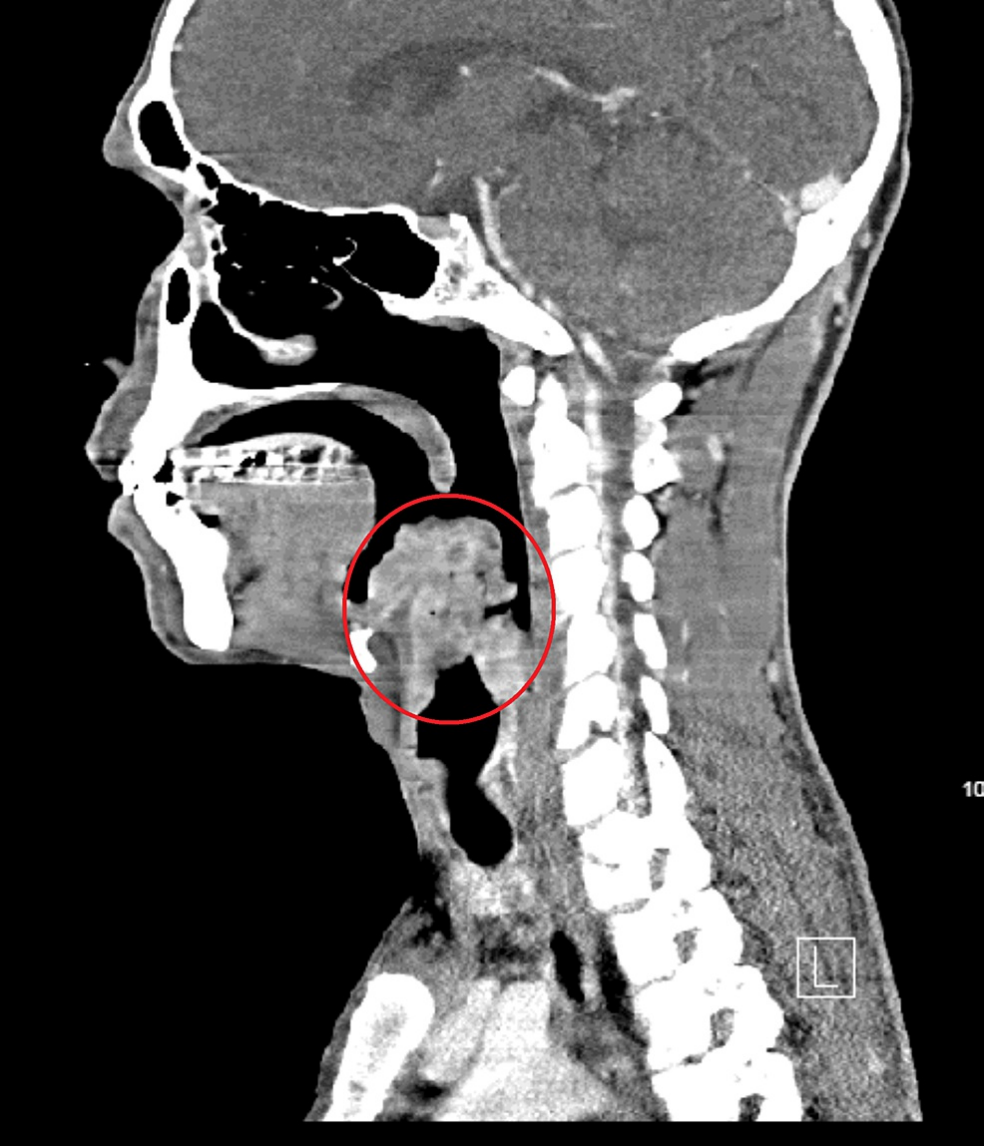
Computed tomography of the soft-tissue neck with contrast sagittal section (lesion in the circle).

CT of the neck demonstrated a lobulated heterogeneous enhancing mass lesion, likely from the epiglottis measuring approximately 4.2 × 3.7 × 6.7 cm in size. The mass was extending superiorly to the superior aspect of the oropharynx and inferiorly to the hypopharynx with involvement of the left aryepiglottic fold and possibly the right aryepiglottic fold along with pre-epiglottic fat and the supraglottic/glottic larynx, effacing the bilateral vallecula and left pyriform sinus. The lesion caused a near-complete obstruction of the upper airway at the level of the supraglottic larynx, posterior to the epiglottis (Figures 2, 3).

**Figure 4:**
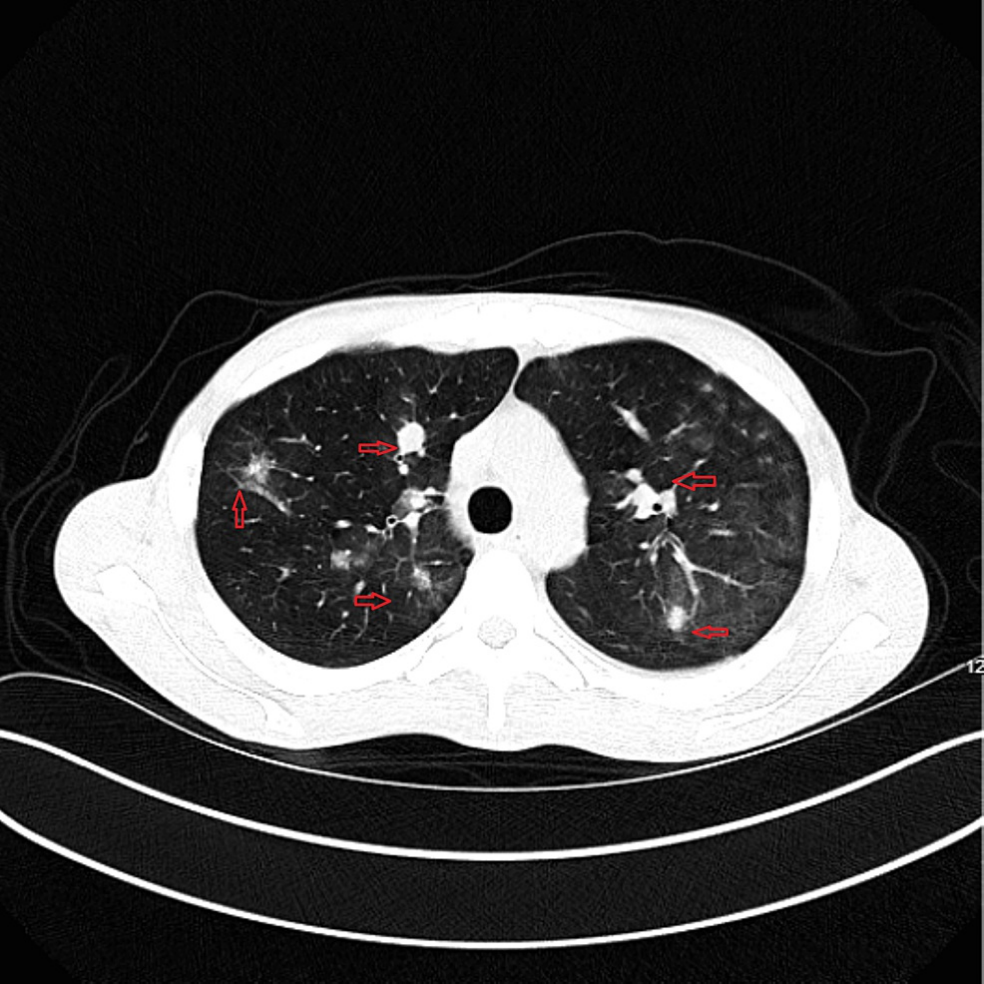
Computed tomography of the chest without contrast. The transverse section indicates ground-glass opacities and other lung lesions (red arrows).

A CT pulmonary angiogram (CTPA) ruled out pulmonary embolism but revealed several bilateral lung nodules with ground-glass opacities concerning for metastases. Hence, a chest CT was done that indicated innumerable, bilateral, randomly distributed lung nodules with multiple patchy and confluent areas of ground-glass opacities, indicating a possible disseminated fungal infection with septic emboli, lymphoma, or metastatic disease (Figure 4). However, no echocardiography was done to rule out vegetation for septic emboli.

**Figure 5:**
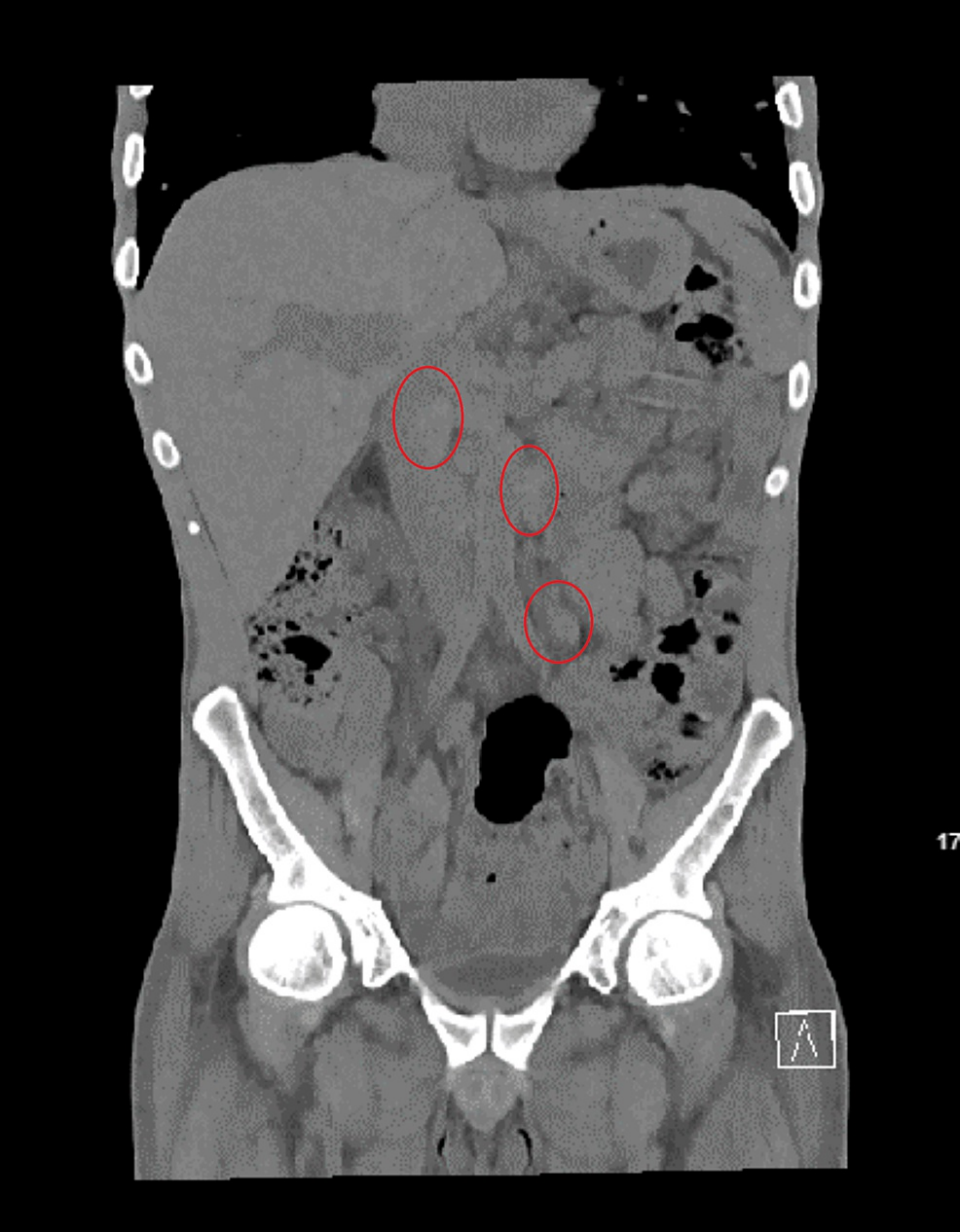
Computed tomography of the abdomen and pelvis without contrast coronal view (lesions in the circle).

A CT of the abdomen further demonstrated upper abdominal and mesenteric adenopathy, with the largest measuring 3 cm, and extensive retroperitoneal adenopathy, with the largest measuring 2.2 cm in the left para-aortic region (Figure 5).

He was started empirically for possible community-acquired bacterial pneumonia with ceftriaxone 2 g daily and azithromycin 500 mg once a day, as well as empiric treatment for pneumocystis with trimethoprim-sulfamethoxazole 20 mg/kg/day divided every eight hours and caspofungin 50 mg every 24 hours. Due to oxygen saturation being less than 90%, 40 mg of daily oral prednisone was initiated for five days with a taper and intravenous (IV) fluconazole 400 mg once daily on day one and then transitioned to 200 mg IV once daily for 14 days for oral candidiasis. Bictegravir-emtricitabine-tenofovir 50-200-25 mg was also started after the patient consented to the treatment.

The large epiglottic mass was causing increased respiratory distress secondary to airway obstruction leading to increased oxygen requirement. An elective tracheostomy was done to prevent respiratory decompensation and percutaneous gastrostomy for nutritional support.

**Figure 6:**
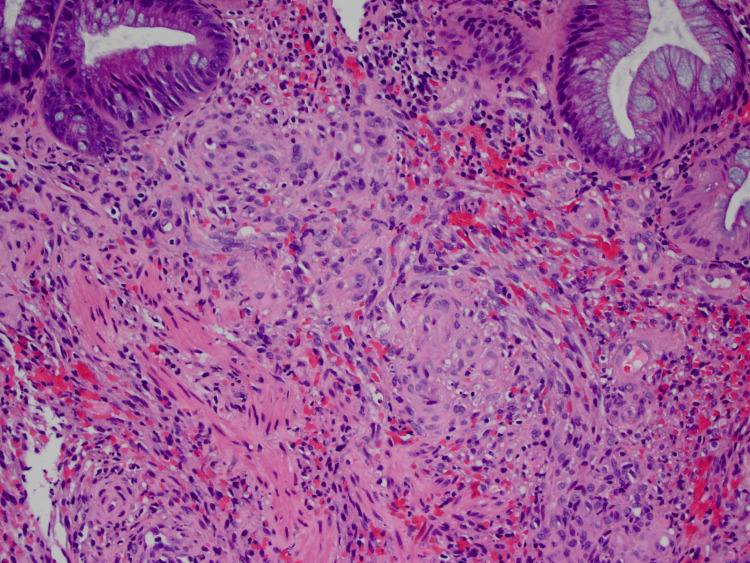
Histopathological image of the retropharyngeal mass (neoplastic spindle cells with numerous intervening blood-filled channels, which is a classic finding in Kaposi sarcoma).

A biopsy was done of the retropharyngeal mass, which was positive for KS. Neoplastic spindle cells were positive for vascular markers CD31 and ERG, and HHV-8, findings consistent with KS. No evidence of lymphoma was found (Figure 6).

For further management, consultation with the oncology department was done for possible radiation therapy to shrink the tumor along with chemotherapy for metastasis to the lungs. In addition, per discussion with the oncology department, the patient was scheduled for a left retroperitoneal lymph node biopsy to aid the staging of the KS. He was counseled regarding the quality of life with the medication and the benefits of chemotherapy and radiotherapy. However, the patient refused any further intervention and refused to undergo chemotherapy and radiotherapy. Because he had decision-making capacities, his autonomy was respected, and he was discharged from the hospital with HAART (bictegravir-emtricitabine-tenofovir) medication only.

## Discussion

Trends of KS and HIV

Dr. Moritz Kaposi originally described KS in 1872 [7]. Over the past 20 years, the incidence of KS in the US population has seen notable variations. Its increased occurrence in the 1980s was unquestionably linked to the start of the AIDS epidemic [8]. The decrease in KS incidence during the 1990s coincided with changes in AIDS incidence due to changes in both the risk of HIV infection and the availability of better treatments. Because KS was extremely uncommon before the AIDS epidemic, the jump in the frequency of this condition was sudden and significant [8]. High-risk populations, such as homosexual males, who are more exposed to HIV/AIDS and KSHV, have dominated the recent KS epidemic. As a result, the AIDS-related KS epidemic that followed during the previous 20 years occurred disproportionately in places like San Francisco, Hawaii, Atlanta, and Seattle, with relatively high significant populations of homosexual men. Contrarily, women suffered a significantly lesser KS epidemic due to their lower frequency of KSHV and likelihood of HIV infection [8-10].

Based on the clinical circumstances in which it develops, KS is classified into four types, namely, classic, endemic, iatrogenic, and AIDS-related (Table 1).

**Table 1:** Types of Kaposi sarcoma. M:F: male-to-female ratio

Type of Kaposi sarcoma	Risk group	Cutaneous	Visceral	Clinical course
Classic sporadic or Mediterranean	M:F prevalence is 3:1 in the Mediterranean or Central/Eastern European origin; Middle East age >60	Yes, limited to distal lower extremities	No	Low malignant potential Survival: years to decades
Endemic (African)	Male adult children of both sexes in equatorial Africa	Yes, various (may be similar to classic or more locally aggressive); lower extremity lymphedema in adults; cutaneous disease often absent in children	Yes, internal organs involved in a subset of adult patients Common (lymph nodes and viscera) in children	Indolent, occasionally aggressive progression with visceral disease Aggressive in children Survival: months to years
Iatrogenic (immunosuppression-related)	Exogenous immunosuppression, especially solid organ transplant; older patients (>50); use of cyclosporin A	Yes, distal lower extremities; may be disseminated	Yes	May heal spontaneously with modification of immunosuppression May be aggressive Survival: months to years
AIDS-related	Men who have sex with men (resource-abundant countries). Heterosexual males and females (Africa)	Yes, localized or disseminated	Yes	Indolent, occasionally aggressive progression May regress with HIV treatment Survival: weeks to months

Pathology

The course of KS lesions has been defined as occurring through three primary pathologic stages [11,12]. Thin-walled vascular spaces with sparse mononuclear cells infiltrating lymphocytes, plasma cells, and macrophages can be seen in the upper dermis during the “patch” stage. The number of vascular gaps increases, the density of the inflammatory infiltrate rises, and spindle cell bundles assemble around the antiproliferative regions in the “plaque” stage. The tumor is more solid and has well-defined nodules in the “nodular” setting composed of sizable fascicles of spindle-shaped endothelial cells with fewer, more tightly packed vascular slits. Some extravasated erythrocytes and macrophages are found between spindle cells, and the mononuclear cell infiltration is no longer prominent [11,12].

Treatment

The majority of the treatment to date has been palliative. The goal of therapy for AIDS-related KS includes symptom relief, KS progression prevention, cosmesis improvement, and reduction of related edema, organ compromise, and psychological stress [13]. Liquid nitrogen, intralesional vinca alkaloids, intralesional interferon alpha, topical imiquimod, radiation, electron beam therapy, photodynamic therapy, and others are among the alternatives for local treatment [7]. Widespread skin involvement (>25 lesions), significant oral KS, noticeable symptomatic edema, fast-progressing disease, symptomatic visceral KS, and KS flares are indications for systemic treatment [7]. The management of AIDS-related KS combines KS therapy with AIDS treatment; HAART is a combination of several drugs used to reduce the damage to the immune system caused by HIV infection, which raises CD4 counts [14]. The preferred regimen usually includes a combination of dual nucleoside reverse transcriptase inhibitors (e.g., emtricitabine) plus protease inhibitors or integrase inhibitors (e.g., bictegravir) and can be used with therapies like interferon alfa [14,15]. Moreover, advanced AIDS-related KS is treated with combined chemotherapy (e.g., daunorubicin, bleomycin, vinblastine) [14,15]. It is a clinical choice whether to use HAART alone or in conjunction with chemotherapy to treat a patient with KS [7].

## Conclusions

Our case highlights the atypical visceral presentation without any cutaneous manifestation of KS in a non-compliant patient diagnosed with HIV. Non-adherence to HAART in patients with HIV may lead to multiple opportunistic presentations, including KS, that may disseminate in different locations, particularly in the airway leading to respiratory failure due to airway occlusion. Therefore, clinicians should not fixate on incidental COVID-19 findings in patients who are non-adherent to HIV medications. In addition, high suspicion is required despite the absence of cutaneous manifestation of KS in HIV patients with low CD4 count in the presence of compromised immune response.

## References

[1] Pantanowitz L, Dezube BJ (2008). Kaposi sarcoma in unusual locations. BMC Cancer.

[2] Nwabudike SM, Hemmings S, Paul Y, Habtegebriel Y, Polk O, Mehari A (2016). Pulmonary Kaposi sarcoma: an uncommon cause of respiratory failure in the era of highly active antiretroviral therapy-case report and review of the literature. Case Rep Infect Dis.

[3] Etemad SA, Dewan AK (2019). Kaposi sarcoma updates. Dermatol Clin.

[4] Dalla Pria A, Pinato DJ, Bracchi M, Bower M (2019). Recent advances in HIV-associated Kaposi sarcoma. F1000Res.

[5] Bray F, Ferlay J, Soerjomataram I, Siegel RL, Torre LA, Jemal A (2018). Global cancer statistics 2018: GLOBOCAN estimates of incidence and mortality worldwide for 36 cancers in 185 countries. CA Cancer J Clin.

[6] Bangar S, Vashisht R, Sonar P (2022). Kaposi sarcoma (KS) with primary effusion lymphoma in HIV infected MSM (men having sex with men) co-infected with pulmonary tuberculosis and syphilis: a case report from India. AIDS Res Ther.

[7] Tan WC, Chan LC (2011). Kaposi's sarcoma: case report and treatment options. Med J Malaysia.

[8] Eltom MA, Jemal A, Mbulaiteye SM, Devesa SS, Biggar RJ (2002). Trends in Kaposi's sarcoma and non-Hodgkin's lymphoma incidence in the United States from 1973 through 1998. J Natl Cancer Inst.

[9] Greenblatt RM, Jacobson LP, Levine AM (2001). Human herpesvirus 8 infection and Kaposi's sarcoma among human immunodeficiency virus-infected and -uninfected women. J Infect Dis.

[10] Lennette ET, Blackbourn DJ, Levy JA (1996). Antibodies to human herpesvirus type 8 in the general population and in Kaposi's sarcoma patients. Lancet.

[11] Grayson W, Pantanowitz L (2008). Histological variants of cutaneous Kaposi sarcoma. Diagn Pathol.

[12] Mentzel T, Knuutila S, Lamovec J (2013). Kaposi sarcoma. World Health Organization Classification of Tumours of Soft Tissue.

[13] Vanni T, Sprinz E, Machado MW, Santana Rde C, Fonseca BA, Schwartsmann G (2006). Systemic treatment of AIDS-related Kaposi sarcoma: current status and perspectives. Cancer Treat Rev.

[14] (2018). Medline Plus. Kaposi sarcoma. https://medlineplus.gov/kaposisarcoma.html.

[15] Schwartz RA, Cohen PJ (1989). Kaposi’s sarcoma. Geriatric Dermatology: Clinical Diagnosis and Practical Therapy.

